# Favorable outcome of SARS-CoV-2 infection in pediatric hematology oncology patients during the second and third pandemic waves in Italy: a multicenter analysis from the Infectious Diseases Working Group of the Associazione Italiana di Ematologia e Oncologia Pediatrica (AIEOP)

**DOI:** 10.1007/s00277-022-04884-x

**Published:** 2022-06-20

**Authors:** Daniele Zama, Francesco Baccelli, Antonella Colombini, Amalia Contino, Elisabetta Calore, Maria Grazia Petris, Linda Meneghello, Federico Mercolini, Andrea Lo Vecchio, Shana Montalto, Cristina Meazza, Angelica Barone, Francesca Compagno, Paola Muggeo, Elena Soncini, Letizia Brescia, Eugenia Giraldi, Nagua Giurici, Rosa Maria Mura, Monica Cellini, Katia Perruccio, Valeria Petroni, Milena La Spina, Ottavio Ziino, Roberta Burnelli, Raffaella De Santis, Maurizio Mascarin, Valentina Barretta, Gloria Tridello, Simone Cesaro

**Affiliations:** 1 Pediatric Oncology and Hematology Unit, IRCCS Azienda Ospedaliero-Universitaria of Bologna, Ospedale Policlinico S. Orsola-Malpighi, Via Massarenti 11, 40138 Bologna, Italy; 2 Department of Pediatrics, Ospedale San Gerardo, University of Milano-Bicocca, 20900 Monza, Italy; 3 Division of Paediatric Onco-Haematology, Stem Cell Transplantation and Cellular Therapy, Città Della Salute E Della Scienza Hospital, 10100 Turin, Italy; 4 Hematology Oncology Division, Department of Women’s and Children’s Health, University of Padova, 35100 Padua, Italy; 5 U.O.M. Pediatria Ospedale S. Chiara, 38100 Trento, Italy; 6 Pediatric Onco-Hematology Unit, Department of Pediatrics, Hospital of Bolzano, 39100 Bolzano, Italy; 7 Department of Translational Medical Sciences, Section of Pediatrics, University of Naples Federico II, 80131 Naples, Italy; 8 Infectious Diseases Unit and COVID-Hospital, IRCCS Istituto Giannina Gaslini, 16100 Genoa, Italy; 9 Pediatric Oncology Unit, Fondazione Istituto Di Ricovero E Cura a Carattere Scientifico, Istituto Nazionale Dei Tumori, 20100 Milan, Italy; 10 Pediatric Onco-Hematology Unit, Pediatric Clinic, Pietro Barilla Children’s Hospital, University of Parma, 43100 Parma, Italy; 11 Pediatric Hematology/Oncology Unit, Fondazione IRCCS Policlinico San Matteo, 27100 Pavia, Italy; 12 Pediatric Oncology-Hematology Unit, Department of Pediatrics, Azienda Ospedaliero Universitaria Policlinico, 7012 Bari, Italy; 13 Pediatric Oncohematology and Bone Marrow Transplant Unit, Children’s Hospital, Spedali Civili, 25100 Brescia, Italy; 14 Hemato-Oncology Unit, SS. Annunziata Hospital, 74100 Taranto, Italy; 15 Pediatric Hematology and Oncology Unit, Papa Giovanni XXIII Hospital, 24127 Bergamo, Italy; 16 Pediatric Hematology-Oncology, Institute for Maternal and Child Health IRCCS “Burlo Garofolo”, 34100 Trieste, Italy; 17 Pediatric Oncology Unit, Azienda Ospedaliera Brotzu, 09100 Cagliari, Italy; 18 Pediatric Oncology-Hematology Unit, Department of Mother and Child, Azienda Ospedaliero Universitaria Modena, 41100 Modena, Italy; 19 Pediatric Oncology-Hematology Unit, Santa Maria Della Misericordia Hospital, 06100 Perugia, Italy; 20 Division of Pediatric Hematology and Oncology, Ospedale G. Salesi, 60100 Ancona, Italy; 21 Pediatric Hematology and Oncology Unit-AOU Policlinico “Rodolico-San Marco”, University of Catania, 95100 Catania, Italy; 22 Department of Pediatric Hemato-Oncology, ARNAS Ospedali Civico, G. Di Cristina, 90100 Palermo, Italy; 23 Pediatric Onco-Hematology Unit, Sant’Anna Hospital, 44100 Ferrara, Italy; 24 Hemato-Oncology Unit, Department of Pediatrics, ‘Casa Sollievo Della Sofferenza’ Hospital, 71013 San Giovanni Rotondo, Italy; 25 Adolescent and Young Adult Oncology and Pediatric Radiotherapy Unit, CRO-Centro Di Riferimento Oncologico Di Aviano, Istituto Di Ricovero E Cura a Carattere Scientifico, 33081 Aviano, Italy; 26 Pediatric Hematology Oncology Unit, Department of Mother and Child, Azienda Ospedaliera Universitaria Integrata, 37100 Verona, Italy

**Keywords:** SARS-COV- 2 infection, Pediatric oncology, Children, COVID-19, Outcome

## Abstract

COVID-19 has a mild clinical course with low mortality rate in general pediatric population, while variable outcomes have been described in children with cancer. Infectious diseases working party of the AIEOP collected data on the clinical characteristics and outcomes of SARS-CoV-2 infections in pediatric oncology/hematology patients from April 2020 to May 2021, including the second and the third waves of the pandemic in Italy. Factors potentially associated with moderate, severe, or critical COVID-19 were analyzed. Of the 153 SARS-Cov2 infections recorded, 100 were asymptomatic and 53 symptomatic. The course of COVID-19 was mild in 41, moderate in 2, severe in 5, and critical in 5 children. A total of 40.5% of patients were hospitalized, ten requiring oxygen support and 5 admitted to the intensive care unit. Antibiotics and steroids were the most used therapies. No patient died due to SARS-CoV-2 infection. Infections occurring early (< 60 days) after the diagnosis of the underlying disease or after SCT were associated to moderate, severe, and critical disease compared to infections occurring late (> 60 days) or during maintenance therapy. In the patients on active chemotherapy, 59% withdrew the treatment for a median of 15 days. SARS-CoV-2 presented a favorable outcome in children with cancer in Italy during the pandemic. Modification of therapy represents a major concern in this population. Our findings suggest considering regular chemotherapy continuation, particularly in patients on maintenance therapy or infected late after the diagnosis.

## Introduction

SARS-CoV-2 infection and the related disease, named novel coronavirus disease 2019 (COVID-19), represent a severe threat to human health with a marked impact on the economic, social, and working human activities on a global scale [1]. The general pediatric population is affected by a milder clinical course with lower rate of hospitalization and mortality. However, severe disease and respiratory or multiorgan failure can occur in pediatric COVID-19 [2, 3]. Furthermore, multi-system inflammatory syndrome of childhood (MIS-C) represents a peculiar clinical presentation of SARS-CoV-2 infection in children with potentially life-threatening course [4]. Children and adolescents with cancer or recipients of stem cell transplantation (SCT) represent a vulnerable population with a well-known higher risk of morbidity and mortality due to community-acquired respiratory viral infections [5]. In adults with cancer, the outcome of SARS-CoV-2 infection appears poorer when compared to general adult population, with higher mortality rate [6, 7]. Pediatric oncology patients showed highly variable outcomes with severe or critical infections ranging from 6 to 20% and mortality ranging from 0 to 10% in small observational studies, mostly performed in high income countries [8, 9]. The Global Registry of COVID-19 in Childhood Cancer (GRCCC) comprises a large multinational cohort of patients from all World Bank income groups reporting a 19.9% of severe or critical disease and a mortality due to COVID-19 of 3.8%. Several clinical features, such as lymphopenia and neutropenia, have been identified as risk factors of severe infection. Interestingly, low-income and lower-middle-income as well as upper-middle-income country status are associated with higher risk of severe or critical disease [10]^.^ The Infectious Diseases Working Group (IDWG) of the Italian Pediatric Hematology Oncology Association network (AIEOP) performed a first description of severity and outcome of SARS-CoV-2 infection in 29 Italian oncological pediatric patients, diagnosed during the first pandemic wave (February–May 2020), reported that only 12 patients were symptomatic, of whom only 3 presented with pneumonia and none presented severe or critical disease or required intensive care admission or died of SARS-CoV-2 infection, whereas the main consequence of the infection was the withdrawal of chemotherapy treatment [11]. Considering the limited time of observation, the little sample size of this first report and the potential change of epidemiology due to the emergence of viral variants, we assessed the characteristics and the outcome of SARS-COV-2 infection during the second and third pandemic waves in Italian pediatric hematology oncology population.

## Methods

The IDWG of AIEOP performed a prospective analysis of the clinical characteristics, treatment, and outcome of patients diagnosed of SARS-CoV-2 infection. AIEOP centers were invited to collect data on infected patients since the start of pandemic (March 2020). The present report shows data of patients with confirmed SARS-CoV-2 infection diagnosed from April 2020 to May 2021. This period includes the second and the third wave of the pandemic in Italy, before the approval of COVID-19 vaccines under 18 years of age and the start of vaccination campaign in Italy for children. The AIEOP-participating centers were 24 and represent the entire national territory (16 centers in the North of Italy, 2 in the Middle, and 6 in the South and Islands). Data collection was performed by each local co-investigator trough an anonymized paper case report form. Patients’ follow-up was updated on August 31, 2021. Patients ≤ 18 years of age were included if they have been treated with chemotherapy and/or radiotherapy irrespective of treatment status, or have received SCT, and were diagnosed of SARS-CoV-2 infection confirmed by molecular (RT-PCR) or antigen testing on nasopharyngeal swabs or bronchoalveolar lavage.

SARS-CoV-2 infections were classified as asymptomatic and symptomatic. COVID-19 was defined by the presence of fever with or without symptoms or signs of respiratory tract or gastrointestinal tract, or any other organ involvement. On the basis of the clinical, laboratory and radiological characteristics, the severity of COVID-19 was graded as mild (upper respiratory or gastro-intestinal symptoms), moderate (acute lower respiratory tract infection without hypoxemia), severe (hypoxia < 94% with or without supplemental oxygen), or critical (need of intensive care unit or organ support), according to published criteria [12].

On patients on chemotherapy, SARS-CoV-2 infection was further classified according to the time of occurrence as early or late episode (within or after 60 days from the diagnosis of the underlying disease, respectively), episode on maintenance, and episode after SCT. Maintenance chemotherapy includes low-medium intensity chemotherapy that follows the phases of induction and consolidation, as expected in the protocols for acute lymphoblastic leukemia and high-risk rhabdomyosarcoma [13, 14].

Blood count values during the infectious episode were also recorded, including white blood cells (WBC), neutrophils, lymphocytes, monocytes, hemoglobin (Hb), platelets (PLT), and lactate dehydrogenase (LDH). Severe neutropenia was defined as absolute neutrophil count (ANC) < 0.5 × 10^9^/L and severe lymphopenia as absolute lymphocyte count (ALC) < 0.3 × 10^9^/L.

Data about management and outcomes were described, including modification of chemotherapy and time to SARS-CoV-2 negativization at nasopharyngeal swab.

The study was approved by the Ethics Committee of the participating centers and informed consent was obtained from parents and/or patients, as appropriate. All procedures followed were in accordance with the ethical standards of the responsible committee on human experimentation (institutional and national) and with the Helsinki Declaration of 1975, as revised in 2008.

### Statistical analysis

The main characteristics of patients were reported by descriptive statistics. Median, minimum, and maximum values were used for continuous variables, while absolute and percentage frequencies were used for categorical variables. Comparisons between categorical variables were performed by the chi-square or Fisher exact test, as appropriate, while continuous variables were compared by *t*-test or Mann–Whitney test. The identification of factors potentially associated with moderate, severe, or critical infection was performed by logistic regression analysis. The factors assessed were age, gender, weight, type of underlying disease, time of occurrence SARS-CoV-2 infection (early, late, maintenance) previous HSCT, severe neutropenia, and antifungal prophylaxis. The median follow-up was computed by using the reverse Kaplan Meier methods. A *p*-value ≤ 0.05 was considered statistically significant. All *p*-values are two-sided. All the analyses were performed using the statistical software SAS v. 9.4 (SAS Institute Inc., Cary, NC, USA).

## Results

During the study period, 153 patients with SARS-COV-2 infection were recorded. The diagnosis was based on positive nasopharyngeal molecular swab in 145 patients, positive nasopharingeal antigen swab in 2 patients while this information was missing in 6 patients. Infection was asymptomatic in 100 children (65.4%) and symptomatic in 53 (34.6%). Table 1 shows the main demographic and clinical characteristics according to presence or not of symptoms. 130/153 (85%) children developed the infection after or during chemotherapy, while 23/153 (15%) previously received SCT (19 allogeneic, 4 autologous). Among the 130 cases occurring after or during chemotherapy, SARS-CoV-2 infection occurred early after diagnosis of the underlying condition in 25 patients (19.2%) and late in 69 (53.1%) patients, respectively, while 35 (26.9%) patients developed the infection during maintenance treatment. Median time of SARS-CoV-2 infection from diagnosis was 220 days (19–2231). In the 23 transplanted patients, immunosuppressive therapy was ongoing in 10 out of 19 patients with SARS-COV-2 infection after allogeneic SCT while no information was available for 4 allo-SCT patients. No significant difference in clinical and demographic features was detected in asymptomatic and symptomatic patients. Among the fifty-three (35%) symptomatic patients, fever was the most common symptom, reported in 40 (75%) children. Other symptoms were diarrhea in 11 (21%), cough in 10 (19%), hypoxemia in 9 (17%), lower respiratory tract symptoms in 8 (15%), vomits in 5 (9%), and hypotension in 2 (4%) patients, respectively. MIS-C was diagnosed in only 1 patient. The disease was classified as mild in 41 (26.8%), moderate in 2 (1.3%), severe in 5 (3.3%), and critical in 5 (3.3%) children, respectively. No patient presented coagulative disorders. Radiological investigation was performed in 33 patients (23 chest X-ray, 5 lung computed tomography (CT) scan, 5 both chest X-ray, and lung CT scan.), and all were symptomatic but one, who underwent chest X-ray. Blood count assessment at the time of SARS-COV-2 infection did not show any significant quantitative difference between asymptomatic and symptomatic patients, except for platelets count and LDH, as shown in Table 2. Data on therapies and outcomes are summarized in Table 3. A total of 40.5% of patients (62/153) were hospitalized, 24/100 asymptomatic and 38/53 symptomatic (*p* < 0.0001) (Table 4). Ten symptomatic patients required O^2^ support and 5 patients were admitted to intensive care unit. Characteristics of these five patients are described in Table 5. Antibiotics and steroids were the most used therapies, particularly in symptomatic patients. Other therapies were antivirals, hydroxycloroquine, polyclonal immunoglobulins, and convalescent plasma. One patient received monoclonal antibodies. In the 98 patients on active treatment, 39/69 (56.5%) asymptomatic and 19 of 28 (67.9%) symptomatic patients withdrew the treatment for a median of 15 days, (range 1–44) and 19 days (range 2–82), respectively (missing data in one patient). Median time to SARS-CoV-2 negativization on NF swab was 20 days (range 1–112), significantly longer in asymptomatic (29 days) compared to symptomatic patients (19 days) (*p* 0.01). After a median follow-up of 2.1 months (95% *CI*: 1.8–2.6), 150 patients were alive. Three patients died due to disease progression (one with relapsed Ewing sarcoma, one with rhabdomyosarcoma in partial remission, and one with undifferentiated rhino pharyngeal carcinoma). No death was related to SARS-CoV-2 infection. No patient experienced SARS-Cov2 reinfection during the study period. The analysis performed to identify possible factors associated with moderate, severe, and critical disease is shown in Table 4. Infections occurring early (< 60 days) after the diagnosis or after SCT were statistically associated to moderate, severe and critical disease compared to infections occurring late (> 60 days) or during maintenance therapy.

**Table 1:** Main demographic and clinical characteristics of patients with SARS-CoV-2 infection. SCT, stem cell transplantation; IS, immunosuppressive therapy

	Asymptomatic, *N*° (%) 100 (65.4)	Symptomatic, *N*° (%) 53 (34.6)	Total, *N*° (%) 153 (100.0)	*p*
Sex M/F				
• F	41 (41.0)	26 (49.1)	67 (43.8)	0.3
• M	59 (59.0)	27 (50.9)	86 (56.2)	
Median age, median (range) (years)	7 (0–17)	10 (0–17)	7 (0–17)	0.2
Median weight, median (range) (kilograms)	24 (8–100)	31 (5–98)	25 (5–100)	0.3
Underlying disease				
• Acute leukemia/lymphoma	59 (59.0)	34 (64.2)	93 (60.8)	0.6
• Solid tumors	33 (33.0)	14 (26.4)	4730.7)	
• Histiocytosis	4 (4.0)	1 (1.9)	5(3.3)	
• Non malignant disease*	4 (4.0)	4 (7.5)	8(5.2)	
Chemotherapy	88 (88.0)	42 (79.2)	130 (85.0)	0.1
• Early (within 60 days from diagnosis)	16	9	2519.2)	
• Late (after 60 days from diagnosis)	45	24	6953.1)	
• On maintenance	26	9	3526.9)	
• Missing	1	0	1 (0.8)	
Time from diagnosis to SARS-CoV-2, median (range) (days)	215 (19– 2231)	248 (19–1692)	220 (19–2231)	0.9
SCT	12 (12.0)	11 (20.8)	2315.0)	
• Allogeneic	9	10	19	
• Autologous	3	1	4	
On IS therapy				
• Yes	3	7	10	
• No	5	4	9	
Time from SCT to SARS-CoV-2 median (range) (days)	298 (77– 3910)	219 (50–1007)	219 (50– 3910)	
Severe neutropenia (neutrophils < 500/mmc) at diagnosis of SARS-CoV-2	16 (16.0)	12 (22.6)	28 (18.3)	0.3
Severe lymphopenia (lymphocytes < 300/mmc) at diagnosis of SARS-CoV-2	45 (90.0)	37 (90.2)	82 (90.1)	1

**Table 2:** Comparison of blood count values in asymptomatic and symptomatic patients at the beginning of SARS-CoV-2 infection, WBC, white blood count; LDH, lactate dehydrogenase

	Asymptomatic, median (range)	Symptomatic, median (range)	Total	*p*
WBC, N × e9/L	3.76 (0.43–424.00)	3.84 (0.22–95.97)	3.77 (0.22–424.00)	0.6
Neutrophils, N × 10e9/L	1.50 (0.04–9.79)	1.72 (0–23.98)	1.64 (0–23.98)	0.6
Lymphocytes, N × 10e9/L	1.00 (0.16–12.74)	1.10 (0.06–21.80)	1.00 (0.06–21.8)	0.9
Monocytes, N × 10e9/L	0.45 (0.01–1.88)	0.41 (0–12.40)	0.44 (0–12.40)	0.7
Hemoglobin, g/dl	11 (7–15.6)	9.9 (4.5–15.0)	10.6 (4.5–15.6)	0.1
Platelets, N × 10e9/L	217.00 (13.00–738.00)	133.00 (2.00–801.00)	184.00 (2.00–801.00)	0.04
LDH, mU/ml	0.26 (0.13–2.09)	0.32 (0.16–12.91)	0.27 (0.13–12.91)	0.03

**Table 3:** Outcome of SARS-CoV-2 infection in the study cohort. SCT, stem cell transplantation

	Asymptomatic, N° (%)	Symptomatic, N° (%)	Total	*p*
Hospitalization	24/100 (24.0)	38/53 (71.7%)	62/153 (40.5)	< 0.0001
Therapy • Antibiotics • Antivirals • Steroids • Hydroxychloroquine • Policlonal Immunoglobulins • Convalescent plasma • Monoclonal antibodies	8/80 (10.0) 2/100 (2.0) 7/96 (7.3) NA 2/97 (2.1) 5/88 (5.7) 0/100	26/50 (52.0) 3/53 (5.7) 17/52 (32.7) NA 2/52 (3.9) 4/53 (7.6) 1/53	34/130 (26.2) 5/153 (3.3) 24/148 (16.2) NA 4/149 (2.7) 9/141 (6.4) 1/153	< 0.0001 0.3 < 0.0001 - 0.6 0.7 -
O2 support	/	10 (18.9)		
Intensive care admission	/	5 (9.4)		
Withdrawal of chemotherapy (SCT patients excluded)	39/69 (56)	19/28 (67)	58/97 (59)	0.3
• Days (range)	15 (1–44)	19 (2–82)	15 (1–82)	0.1
Time to SARS-CoV-2 negative NF swab, days (range)	29 (5–107)	19 (1–112)	20 (1–112)	0.01

**Table 4:** Factors associated to moderate, severe, or critical disease

	Asymptomatic or mild *N*° (%) (*N* = 141)	Moderate, severe or critical *N*° (%) (*N* = 12)	Total (*N* = 153)	*p*
Sex				
• M	79/86 (91.9)	7/86 (8.1)	86 (56.2)	0.9
• F	62/67 (92.5)	5/67 (7.5)	67 (43.8)	
Underlying disease				
• Acute leukemia/lyymphoma	85/93 (91.4)	8/93 (8.6)	93 (60.8)	0.3
• Solid tumors	45/47 (95.7)	2/47 (4.3)	4730.7)	
• Histiocytosis	4/5 (80.0)	1/5 (20.0)	5(3.3)	
• Non-malignant disease	7/8 (87.5)	1/8 (12.5)	8 (5.2)	
Median age, median (range)	7 (0–17)	8.5 (1–16)	7 (0–17)	0.6
Median weight, median (range)	25 (5–100)	30.5 (10–70)	25 (5–100)	0.9
HSCT				
• Yes	20/23 (87.0)	3/23 (13.0)	23 (15.0)	0.4
• No	121/130 (93.1)	9/130 (6.9)	130 (85.0)	
Time of SARS-CoV-2 infection occurrence				
• Early	20/25 (80.0)	5/25 (20.0)	25 (16.5)	0.04
• Late	66/69 (95.7)	3/69 (4.3)	6945.4)	
• On maintenance	34/35 (97.1)	1/35 (2.9)	3523.0)	
• After SCT	20/23 (87.0)	3/23 (13.0)	23 (15.1)	
Severe neutropenia (PMN < 500) at diagnosis of SARS-CoV-2				
• Yes	24/28 (85.7)	4/28 (14.3)	28 (18.3)	0.2
• No	117/125 (93.6)	8/125 (6.4)	125 (81.7)	
Antifungal prophylaxis				
• Yes	15/17 (88.2)	2/17 (11.8)	17 (11.1)	0.6
• No	126/136 (92.6)	10/136 (7.4)	136 (88.9)

**Table 5:** Clinical characteristics of patients admitted to ICU. HLH, hemophagocytic lymphohistiocytosis; ALL, acute lymphoblastic leukemia; HFNC, high-flow nasal cannula; NIV, non-invasive ventilation

Age (years)	Sex	Weight (kg)	Underlying diagnosis	On chemotherapy	SCT	Neutrophils < 500/mmc	Chemotherapy withdrawal	Symptoms	Ventilation support	Kidney function support	Follow-up
14	F	41	aplastic anemia	no	Yes	Yes	N.A	Hypoxemia, pneumonia, fever	HFNC	No	Alive
3	M	20	ALL, first diagnosis	induction	No	No	Yes (24 days)	Hypoxemia, pneumonia, fever, cough	HFNC, NIV	No	Alive
1	M	10	HLH	No	Yes	No	N.A	Hypoxemia, pneumonia, fever	Mechanical ventilation	Yes (diuretics)	Alive
6	F	17	Medulloblastoma	No*	No	No	No	Hypoxemia, pneumonia, fever, shock	NIV	No	Alive
4	F	13	ALL, first diagnosis	consolidation	No	Yes	Yes (20 days)	Hypoxemia, pneumonia, fever, diarrhea, MIS-C	HFNC, NIV	No	Alive

## Discussion

We herein described the prospective data collection of AIEOP-IDWG regarding the clinical characteristics and outcome of SARS-CoV-2 infections in pediatric hematology oncology patients during the second and third wave of the pandemic in Italy. We reported a favorable outcome of the infection in this category of patients, similarly to the first wave description performed by our group [11].

We prospectively enrolled 153 patients from April 2020 to May 2021. To our knowledge, this is the largest multicenter single-country cohort of pediatric cancer patients with SARS-CoV-2 infection described so far. We collected data from the entire national territory and started the enrollment since the very first phase of the pandemic. Together with the adoption of preventive measures recommended by national health authorities, we initiated a screening program by nasopharyngeal swab for all pediatric cancer patients before allowing them to enter hospital to start chemotherapy or undergoing supportive therapies since February 2020 [15]. Furthermore, national and international pediatric oncology associations provided a rapid global response for children with COVID-19 with specific recommendations in order to assure the best clinical management of this particular category of patients [16]. All these measures increased the homogeneity of the collected data.

Italy was the first Western country to deal with COVID-19 outbreak in 2020 and concurrently the first one to adopt strict measures in order to limit the spread of the infection [17]. During the first wave of the pandemic, we described a general positive outcome of SARS-CoV-2 infection without any severe or critical case in children with cancer, similar to general pediatric population [11]. In the present report, we expanded the cohort of patients and confirmed this favorable clinical course, even considering the change in the epidemiology of the pandemic and the emerging of new viral variants [18, 19].

In our cohort of 153 children, only 10 (6.6%) suffered from a severe or critical form of COVID-19, while the vast majority of patients was asymptomatic or presented only mild symptoms. Ten patients required oxygen support and five were admitted to intensive care unit. No death was related to SARS-CoV-2 infection during the entire observational period. The Global Registry of COVID-19 in Childhood Cancer (GRCCC) was created at the beginning of the pandemic and collected data from the global community in order to describe disease severity and identify factors associated with severe SARS-CoV-2 infection in pediatric cancer patients. Severe or critical illness was reported in 259/1319 patients (19.9%) with a mortality rate of 3.8%, higher than general pediatric population. Intensive treatment, low ANC and ALC, and World-bank income country status were factors associated to disease severity [10]. Similar outcomes were described in another international multicenter study reporting data about 131 patients from ten different countries [20]. Variable outcomes were described in other smaller single-center studies conducted in high-income Western countries [8, 21–23]. This variability was also highlighted in reports from low-middle income countries [24, 25]. Generally, the percentage of severe and critical infections ranges from 6 to 20% with a mortality rate from 0 to 10% in different reports [9, 26].

The only factors associated to the presence of symptoms in our cohort were low platelets count and higher LDH levels. The latter could be expression of a stronger inflammatory response related to the infection. Data about platelet count were not available in other studies, while we did not found an association of symptomatic or moderate, severe or critical disease with other blood test values like low ANC and ALC, differently from other reports [10, 20].

We showed that the occurrence of the infection early after the underlying disease diagnosis in patients receiving chemotherapy represents a risk factor for moderate, severe, or critical disease. Also, recipients of SCT presented a higher risk of moderate to critical clinical course. These findings partially confirms data from the global cohort in which patients on active treatment or transplanted presented a higher risk of developing a severe or critical infection, possibly related to a significant immunosuppression level and/or a higher susceptibility to respiratory complications [10]. In our analysis, we also included moderate forms of the disease considering the potential impact of acute lower respiratory tract infections in pediatric patients with cancer, even without hypoxemia and need of oxygen support. Of note, we did not collected data about possible concurrent bacterial coinfections, described as factors associated with COVID-19 severity in other reports [20].

We confirmed that a high percentage of patients underwent therapy withdrawal, as already shown in our previous report [11]. This could possibly affect the prognosis of these patients and represents a serious concern of the pediatric oncological global community [27]. Future studies are needed in order to investigate the potential impact of the pandemic on therapy delay and access to clinical trials. It could also be of interest to describe the possible modification in the percentage of patients discontinuing chemotherapy during the different phases of the pandemic. Interestingly, the withdrawal occurred in the majority of both symptomatic and asymptomatic patients. In the above mentioned global-cohort study by Mukkada and colleagues, the factors associated to treatment modification were different to those associated to disease severity and include upper-middle-income country status [10]. The mild course of the disease described in our cohort suggests to consider regular protocol continuation, particularly in patients on maintenance therapy or infected late after diagnosis of the underlying disease. Previously published national and international recommendations suggest cancer-directed therapy continuation, particularly in hematological malignancies [8, 16].

It has to be considered that, even if general pediatric population is known to be affected by a milder clinical course of COVID-19, children can be affected by peculiar severe forms, particularly MIS-C [28], that, interestingly, was described in only one patient of our cohort. This may suggest a limitation of the intensity of the inflammatory phase of COVID-19 in pediatric oncology hematology patients. Furthermore, immunosuppressed children can develop particular immune-mediated hematological manifestations and coagulative disorders due to SARS-CoV-2 infections, as described in several reports [29, 30]. These clinical features can represent a relevant issue in children with cancer and further studies are needed in order to better understand this field.

## Conclusion

We reported a favorable outcome of SARS-CoV-2 infection in pediatric oncology and hematology patients during the first three pandemic waves in Italy. Nevertheless, we showed modification of cancer-directed therapy as a relevant effect of COVID-19 in this category of patients, highlighting the importance of prevention of the infection as well as the need of recommendations in order to standardize the approach to COVID-19 management and chemotherapy protocols continuation. Furthermore, the feasibility and efficacy of vaccination need to be emphasized, also in this fragile category of patients [31]. Our group recently published a consensus document that aims to help clinicians regarding vaccination approach to these patients, in order to prevent treatment delays and to permit the regular management of pediatric oncological diseases during the pandemic [32].
